# Development, content validation and piloting of a questionnaire on how midwives in Germany provide advice on early childhood allergy prevention in a health literacy responsive way

**DOI:** 10.18332/ejm/215910

**Published:** 2026-02-18

**Authors:** Julia von Sommoggy, Barbara Fillenberg, Laura-Sophie Reitberger, Jonas Lander, Maja Pawellek, Christian Joachim Apfelbacher, Susanne Brandstetter

**Affiliations:** 1 Institute of Social Medicine and Health Systems Research, Medical Faculty, Otto-von-Guericke University Magdeburg, Magdeburg, Germany; 2 Medical Sociology, Department of Epidemiology and Preventive Medicine, Regensburg University Medical Centre, Regensburg, Germany; 3 University Children's Hospital Regensburg, Hospital St. Hedwig of the Order of St. John, University of Regensburg, Regensburg, Germany; 4 Member of the Research and Development Campus Regensburg, Hospital St. Hedwig of the Order of St. John, Regensburg, Germany; 5 Midwifery Science, Medical Faculty, Johannes Gutenberg University Mainz, Mainz, Germany; 6 Institute for Epidemiology, Social Medicine and Health Systems Research, Hannover Medical School, Hannover, Germany; 7 Family Medicine and Primary Care, Lee Kong Chian School of Medicine, Nanyang Technological University, Singapore, Singapore

**Keywords:** health literacy, early childhood allergy prevention, midwives, questionnaire development, content validation

## Abstract

**INTRODUCTION:**

Preventive behaviors in the first three years of life may reduce the onset of allergic conditions. Midwives support families closely during this time and hence could play a key role in strengthening parental health literacy regarding early childhood allergy prevention. The aim of this study was to develop, content-validate and pilot a questionnaire to improve the currently low level of evidence on practices, barriers and facilitators of providing advice on early childhood allergy prevention in a health literacy responsive way by midwives in Germany.

**METHODS:**

We developed a 64-item online questionnaire informed by the findings of a previous qualitative study. Subsequently, the content of the questionnaire was tested in cognitive interviews with midwives and public health experts. The focus was on: overall impression, comprehensibility, response options, relevance, completeness, and ideas for improvement. Then, two versions were piloted in two German federal states on acceptability and to learn more about recruiting midwives for research.

**RESULTS:**

Data from the cognitive interviews (n=8) and the piloting (n=59) indicated that the questionnaire is understandable, feasible and relevant for the target group. Suggestions for improvement focused mainly on midwifery specific terms. The ‘no answer’ option was considered important for all questions. Response options appeared appropriate and scales were mostly fully used.

**CONCLUSIONS:**

Following minor adaptions, the questionnaire can now be applied on a larger scale, as a nationwide survey in Germany addressing all midwives. In order to reach midwives to participate in research, a multifaceted but personal approach seems advisable.

## INTRODUCTION

More than 20% of the German population suffers from allergies, including allergic rhinitis, food allergy, asthma and atopic dermatitis^1-3^. These non-communicable diseases appear to be increasing worldwide and can significantly affect health and well-being. They are representing a major public health concern^4-8^.

Research indicates that the risk of allergies can be reduced during the first three years of life by certain health behaviors^9^, e.g. breast feeding, introduction of complementary feeding between four and six months of age while continuing to breastfeed. These topics are typically covered by midwives. However, evidence has shifted significantly in this field. Recommendations changed from avoidance of allergens during the first year of life to early exposure, to reduce the risk of allergies^10^. Evidence on the effects of interventions for early childhood allergy prevention (ECAP) remains inconclusive and evolving, which makes it difficult to keep up to date with the recent recommendations^11,12^.

Health professionals can be particularly important when it comes to providing information and explaining changing evidence and alterations in recommendations. Midwives are in a unique position to offer guidance during a time in which ECAP needs to be addressed. Postnatal care by midwives in Germany is widely provided in the homes of families. After giving birth, mothers are entitled to up to two home visits a day for the first ten days, followed by further 16 visits by the midwife during the first 12 weeks, and eight more up until the end of the ninth month^13^. They closely support families at a vulnerable time of transition, when ECAP measures could be applied. It is therefore important to ascertain whether and how midwives provide advice on ECAP.

The Health literacy (HL) as the competence to access, understand, appraise and apply health-related information^14^ of parents and patients, needs to be considered when providing information. The consideration of parental HL entails the assessment of parental HL and the application of HL-responsive strategies, i.e. supporting them in accessing, understanding, appraising and applying health-related information. This can include the use of visual materials to support explanations, omitting medical terms and using easy language to facilitate understanding or applying the teach-back technique, to ensure parents have really understood the information provided. In summary, our understanding of HL-responsive advice comprises the communication of evidence-based health information in a way that enables people to understand, appraise, and apply this information with a view to engaging and supporting them in making health-related decisions.

As, to our knowledge, there was no study focusing on how midwives in Germany provide advice on ECAP, we performed an exploratory qualitative study with 24 midwives to gain insight into how midwives inform themselves about ECAP and how they consider and address HL when providing advice on ECAP^15^. Our results indicated that midwives were aware of having a window of opportunity to provide advice on ECAP and also perceived it as their task. They were aware of the current recommendations; however, the national guideline on allergy prevention was unknown to most. Also, they stated to inform parents only implicitly about ECAP by talking about nutrition, hygiene, use of cosmetic products and smoking. Additionally, our results indicated that the concept of HL was unknown to most of the midwives. The assessment of parental HL was described to be based on gut feeling and intuition, as well as information on parental education and employment. None of the midwives used formal strategies to assess parental HL. As recommendations in regard to allergy prevention were perceived as easy to understand, specific strategies to provide advice in a HL-responsive way were not used. When we asked if the teach-back technique was used, it was rather rejected as it was unclear how to apply this technique sensitively.

At present, there remains a paucity of knowledge regarding midwives’ practices, but also barriers and facilitators of providing information on ECAP in a HL-responsive way on a larger scale. Therefore, the aim of this study was to develop, validate and pilot a questionnaire based on our qualitative insights, to assess current practices, facilitators and barriers of providing advice on ECAP in a HL-responsive way.

## METHODS

This is a methodological study for the development of a questionnaire. The questionnaire was developed, validated and piloted in a multi-stage approach. It is meant to capture the following constructs: practices, barriers and facilitators of midwives providing advice on ECAP in a HL-responsive way. We included questions on needs and wishes to support the provision of advice on ECAP in a HL-responsive way, as the development of an intervention is intended in the future. The questionnaire is based on a literature review and the results of our qualitative study^15^. We defined categories and subsequently formulated items, which were then tested for content-validity in cognitive interviews with experts and midwives. The interviews were based on the recommendations of the Consensus-based Standards for the Selection of Health Measurement Instruments (COSMIN) on aspects of content validity – relevance, comprehensibility, and comprehensiveness^16^. Finally, the questionnaire was piloted to ensure that it would be acceptable to the target group, that scales and response options were appropriate, and the questions were correctly understood, e.g. distinguishable from each other. The study received ethical approval from the Ethics Committee of the University of Regensburg (Approval number: 18-1205-101; Date: 21 November 2018). Participants provided informed consent prior to study participation.

### Item development

*Literature review*

We first performed a literature review to identify similar approaches and existing instruments focusing on midwives and/or other health professionals providing advice on health-related topics in a HL-responsive way in their daily practice. However, we found only one instrument related to our aim and target group. The cross-sectional survey by Creedy et al.^17^ focuses on self-reported knowledge and skills of midwives to assess and promote maternal HL. From this, we adapted and included items focusing on midwives’ skills in regard to providing advice in a HL-responsive way, but excluded knowledge items, as these were either country specific or did not refer to ECAP.

*Qualitative study*

There is little knowledge on midwives’ current practices in regard to ECAP and HL-responsive advice on this topic. Thus, we extracted these from the results of our qualitative study.

To develop items on barriers and facilitators of HL-responsive ECAP advice by midwives, we used the Cabana Framework^18^ and the Theoretical Domains Framework (TDF)^19^ to structure our qualitative findings^15^. The Cabana Framework focuses on barriers regarding the adherence to clinical practice guidelines, which is in line with our aim to understand why the national guideline on ECAP is not being applied and why HL-responsive strategies are not administered. The TDF focuses on identifying determinants of current and desired behaviors, which helps to understand underlying reasons for not providing advice on ECAP in a HL-responsive way. Discussing the relations between our qualitative results and the frameworks within the research team (JvS, LSR, SB) we identified the categories most relevant to our research and excluded those that were not addressed in the interviews or did not appear relevant (Table 1), to keep the questionnaire as concise and focused as possible. We excluded the categories *Lack of self-efficacy* and *Inertia of previous practice* from the Cabana framework. From the Theoretical Domains Framework, we excluded or subsumed the domains *Beliefs about capabilities; Nature of behaviors; Motivation and goals; Memory, attention and decision processes; Emotion and behavioral regulation*. Table 1 provides an overview of the categories in the questionnaire, indicating from which framework the category was derived and which categories were omitted.

**Table 1:** Overview of the overarching categories that served as basis for the item development and from which framework (Cabana Framework^18^ and Theoretical Domains Framework^19^) they were derived.

*Categories*	*Cabana*	*TDF*
**Practice of providing advice on ECAP in a HL-responsive way**		
**Barriers and enablers of providing advice on ECAP in a HL-responsive way**		
Lack of awareness, familiarity/knowledge	x	x
Lack of agreement	x	
*Lack of self-efficacy*	*x*	
*Beliefs about capabilities*		*x*
Lack of outcome expectancy/beliefs about consequences	x	x
Inertia of previous behavior		x
Nature of behaviors		x
External barriers/environmental context and resources, social influences	x	x
Skills		x
Social role and identity		x
Motivation		x
*Memory, attention and decision processes*		*x*
*Emotion*		*x*
*Behavioral regulation*		*x*
**Needs and wishes**		
**Sociodemographic data**		

The categories in italics were excluded or subsumed.

*Formulation of items*

The team (JvS, LSR) actively involved in item formulation started developing items, while frequently revisiting the research question, reviewing literature and consulting several times with experts (midwives and experts in questionnaire design) in meetings (in person and online), presenting the items and discussing them^20^.

The questionnaire was structured in four main parts: 1) current practices of midwives providing advice on ECAP in a HL-responsive way, 2) barriers and facilitators of HL assessment and providing advice in a HL-responsive way, 3) needs and wishes regarding the support of providing advice in a HL-responsive way, and 4) sociodemographic data (see Table 1). We generated an initial list of 78 items in German language, which was reduced to 64 items following ongoing and iterative discussions within the research team (JvS, LSR, MP, SB). These 64 items were converted into an online questionnaire using LimeSurvey^21^. We mainly used a 5-point Likert scale, as well as dichotomous yes and no answers, open-ended questions, and multiple choice items.

### Content validation - cognitive interviews

To ensure the content validity of the questionnaire, we conducted cognitive interviews via an online video platform^22,23^. We recruited experts in ECAP and HL (n=3) as well as midwives (n=5)^16^. Experts on ECAP and HL were recruited from the HELICAP research group (“Health Literacy in Early Childhood Allergy Prevention” funded by the German Research Foundation) and personal contacts within the research group. Midwives were recruited with the help of midwives working at the Regensburg University of Applied Sciences, as well as the Coordination Center of Midwives in Regensburg, Bavaria. We focused on including midwives with Bachelor’s (n=1) and/or Master’s degrees (n=3), to ensure that participants have received basic training in scientific research. The interviews lasted an average of 45 minutes. The mean age of the participants was 45 years (for further information on the participants, see Table 2).

**Table 2:** Sociodemographic data of participants in the cognitive interviews in 2023

*Profession*	*Expertise*	*Highest qualification*	*Age (years)*	*Gender*	*Interview duration (minutes)*
Teacher of midwifery/ midwife	Midwifery	Bachelor	42	Female	32
Research associate	Public Health	PhD	36	Male	46
Research associate	Psychology	Master	30	Female	46
Research associate	Medical Doctor	Master	37	Male	48
Midwife	Midwifery	Master	39	Female	46
Midwife	Midwifery	Master	55	Female	41
Midwife	Midwifery	Master	65	Female	40
Midwife	Midwifery	Professional Education	60	Female	65

We conducted the interviews in a two-step approach. After a short introduction, we applied the think-aloud method^23^, meaning the participants were asked to express their thoughts while filling in the questionnaire. The interviewer (JvS) only reminded the participants to express their thoughts, but did not probe or follow up with questions. After the questionnaire was filled in, we switched from think-aloud to intensive probing and the participants were asked further questions about the questionnaire. The interview guide focused on: 1) Overall impression, 2) Comprehensibility, 3) Response options, 4) Relevance, 5) Completeness, and 6) Suggestions for improvement (Supplementary file Material 1). The interviews were audio recorded, transcribed and subjected to content analysis^23^. The questionnaire was adapted according to the findings from the cognitive interviews.

### Piloting

The questionnaire was piloted in a cross-sectional survey with a convenience sample in two German states. Saarland was chosen as a location to pilot the questionnaire, as it has the smallest association of midwifery in Germany. As we are planning a nationwide survey of Germany, we wanted to conduct the pilot study, to test and verify the questionnaire and the recruitment strategy, on a small sample of midwives. As the return rate was too low, we extended it to Berlin. In contrast to Saarland, Berlin is an urban region with a larger midwifery association. The midwifery associations of Saarland (n=262 members) and Berlin (n=1020 members) sent out the invitation to participate to its members (Saarland: 20 March to 15 April 2024 via E-Mail; Berlin: 12 June to 15 July 2024 via Newsletter). In Berlin, the association also disseminated the call via Instagram. Two reminder emails were sent out in Saarland.

We piloted two versions of the questionnaire. As the focus of our study is to learn more about HL-responsive advice by midwives, we wanted to gain further insight into the understanding of HL by means of an open-ended question. However, it was feared that an open-ended question about a complex definition might either make participants refuse to answer the questions or provide rather diverse answers, thus causing incoherence of the data. The research team therefore decided to pilot two versions of the questionnaire, one with an open-ended question asking the participants to provide the definition of HL, one with the definition of HL following Sørensen et al.^14^, and a 5-point Likert scale asking if this definition was known to the participants^20^.

Once the recruitment for the pilot study was concluded, data were extracted and we conducted an analysis using descriptive statistics with SPSS (IBM Corp. Released 2019. IBM SPSS Statistics for Windows, Version 29.0.2.0 Armonk, NY: IBM Corp). We calculated the mean and standard deviation and examined the response distributions for floor and ceiling effects as well as response bias patterns. To check internal consistency, we calculated Cronbach’s alpha.

### Informed consent and confidentiality

Participation in the cognitive interview study was only possible after providing written informed consent for participation. Completion of the questionnaire was anonymous. A data protection declaration was provided at the beginning of the questionnaire and the information had to be read and accepted by ticking a box. Data storage and handling of personal information followed the data protection policy of the Department of Medical Sociology at the University of Regensburg.

## RESULTS

### Qualitative assessment of content validity using cognitive interviews

The content analysis focused on the main topics of the interview guide mentioned above (see Supplementary file Material 1 for interview guide, and Table 2 for further information on participants).

*Overall impression*

All interviewees expressed a positive impression of the questionnaire. They deemed its length adequate and the layout appealing. The first version of the online questionnaire was colored green, which we changed to blue to avoid visual barriers. The online version worked well on all devices. Midwives in our sample stated that the questionnaire motivated them to reflect on their professional practice, which they described as inspiring and helpful:

*‘I haven’t thought much about allergies. This study encourages me to read more about this topic.’*

*Comprehensibility*

Overall, the questionnaire, instructions, and items, were perceived as mostly easy to understand by all participants. When interview partners hesitated during the think-aloud process, they usually reflected on their professional experience or on the specific wording of the item and how this could be improved. Most participants recalled experiences and memories to specific situations in order to give a coherent response and felt able to answer the questions. There was no item that was considered incomprehensible; however, there were suggestions for improvement, e.g. wording of items, rephrasing of questions, omission of lengthy descriptions and sometimes a different order of the items was suggested:

*‘The description of health literacy as access, understand, appraise and apply, should not be included in all items. We know the definition by now and it is distracting to read it every time.’*

*Response options*

Although all respondents provided answers to all questions during the interviews, they asked to include a ‘no answer’ response option for all items. This was particularly important, as it is not possible to complete the questionnaire without providing answers to all items:

*‘It is important to have the option of not answering all the questions. Otherwise you might feel pressured.’*

Otherwise, the response options were considered easy to understand and relevant to the items.

*Relevance*

All items were considered relevant by the participants and, according to them, relevant aspects of the midwives’ professional life in relation to ECAP and HL were covered by the questionnaire. Midwives found providing advice on vaccinations in relation to ECAP relevant; however, they rejected this item as it was attributed to pediatricians:

*‘I’m not going into vaccinations. This topic is too hot. I leave this to the pediatrician.’*

Further, intuition and experience were considered relevant to assess parental HL; however, this item was also rejected as it was perceived as superordinate to the other items (first impression of the family, questions asked by families, etc.):

*‘My first impression of the family is based on my intuition and experience. I think every midwife will tick “always” for this question.’*

*Completeness*

In general, the questionnaire was mostly considered to be complete. However, the items regarding topics considered important when advising parents on ECAP were reflected quite extensively by the midwives. Some argued for more detailed items, e.g. ‘hygiene’ was suggested to relate not only to room hygiene and body hygiene, but to other aspects, as well, including diapers, clothing, washing, air refresher, etc. However, in order not to make the questionnaire any longer, no further items were developed, instead the topics addressed by the term ‘hygiene’ were made explicit in brackets.

*Suggestions for improvement*

After completing the questionnaire, we asked the participants if they had any suggestions for improving the questionnaire. The only idea that occurred in addition to the suggestion previously mentioned, was to include a 'Thank you' message at the end and to provide further information on the research group. The final, content validated instrument consisted of 64 items including sociodemographic items.

### Quantitative assessment

We received a total of 73 questionnaires (overall response rate: 5.7%; Saarland: 13%; Berlin: 4%); 14 persons only opened the questionnaire and did not fill in any information. They were therefore excluded from our analysis. Of the remaining 59 questionnaires, 7 were partially completed and 52 were fully completed.

The sociodemographic data show that midwives participating in this pilot study were diverse regarding age, professional education and experience (see Table 3). Regarding education, midwives from Berlin were more likely to have a Bachelor’s degree, whereas midwives from Saarland were more likely to have vocational training.

**Table 3:** Sociodemographic data of participants in the questionnaire pilot in Saarland and Berlin in March and July 2024 (N=52)

*Characteristics*	*n (%)*
**Age** (years)	
21–25	2 (3.8)
26–30	7 (13.5)
31–35	11 (21.2)
36–40	7 (13.5)
41–45	3 (5.8)
46–50	5 (9.6)
51–55	9 (17.3)
56–60	5 (9.6)
61–65	3 (5.8)
**Work as a midwife** (years)	
<5	7 (13.5)
6–10	26 (50.0)
16–25	6 (11.5)
26–35	13 (25.0)
Employment status*	
Self-employed	34 (65.4)
Employed	7 (13.5)
Employed with self-employed secondary employment	10 (19.2)
No answer	3 (5.8)
**Highest professional qualification/degree**	
**Vocational training**	30 (57.7)
**Bachelor‘s degree**	15 (28.8)
Master‘s degree	7 (13.5)
Doctorate	0 (0.0)
Habilitation	0 (0.0)
No answer	0 (0.0)
Fields of activity*	
Prenatal care – clinical	6 (115)
Prenatal care – non-clinical	38 (73.1)
Obstetrics – clinical	18 (34.6)
Obstetrics – non-clinical	10 (19.2)
Postnatal care – non-clinical	47 (90.2)
Postnatal ward	3 (5.8)
Preparation for birth	23 (44.2)
No answer	2 (3.8)
Other	6 (11.5)

* Multiple selection possible.

*Acceptability*

Except for seven questionnaires – terminated after the fifth question and the first page change – all questionnaires were complete. The ‘no answer’ option was rarely used, for most items only by one or two respondents (Supplementary file Material 2). Only for two items regarding the agreement with statements on allergy prevention (Supplementary file Material 2, question 4) five participants chose the ‘no answer’ option. For question 7, ‘I know the exact content of the national allergy prevention guideline’ 4 participants chose ‘no answer’. We received no question about how to complete the questionnaire and no negative comments after completion.

*Appropriateness of the response options*

For questions on a 5-point Likert scale, the response options were mostly fully used. Only the questions pertaining to breastfeeding: Q3/1 mean=4.9 (SD=0.4), Q4/1 mean=4.8 (SD=0.6), and smoking Q3/11 mean=4.7 (SD=0.6), received very high scores with minimal standard deviation (Supplementary file Material 2). Even though these items show a ceiling effect, they were kept in the questionnaire for completeness of topics.

We piloted two different versions asking for the definition of HL (Supplementary file Material 2: Q5), one with a closed question on a 5-point Likert scale (‘Are you familiar with the following definition of health literacy’) and the other with an open-ended question (‘I understand health literacy as …’). A total of 47 participants responded to the questionnaire with the closed question and five terminated the questionnaire afterwards. In contrast, 12 participants received the version with the open question and two terminated the questionnaire afterwards. The responses to the open-ended question were highly heterogeneous ranging from ‘Having acquired the knowledge and experience to inform and advice on this topic’ to ‘Knowledge about your own health’. After discussion within the research group, it was decided to eliminate the open-ended question due to the heterogeneity of the responses and to continue with providing the definition.

Further open-ended questions, focusing on the needs and wishes of midwives, were answered by approximately half of the participants. However, no participant terminated the questionnaire due to an open-ended question.

The items assessing wishes for further training in HL (Supplementary file Material 2: Q15) or ECAP (Supplementary file Material 2: Q16) and information to be passed on to parents, were presented as multiple-choice options, which were utilized by the majority of participants. A minority of the participants chose not to respond: Q15 n=3, Q16 n=2, Q17 n=1, selecting the option ‘none of the above’ (Supplementary file Material 2). The option to provide a self-formulated answer was only used on a single occasion in Q17 (Supplementary file Material 2).

*Comprehensibility*

The questionnaire appears to be comprehensible, as we received no further questions from participants. However, items 3 and 4 in question 8 (Supplementary file Material 2) appeared to be unclear. Q8 addresses the question of how to provide advice on ECAP. Items Q8-3 and Q8-4 were designed to be exclusive to each other. A cross-tabulation confirmed that the items were not co-variant; however, the resulting value was low and negative (cov = -0.2), which indicates that the questions were not perceived to be exclusive to each other by all respondents. The questions were rephrased.

*Internal consistency*

Cronbach’s alpha for the constructs ‘Importance of topics in consultations on ECAP’ (11 items, five-point Likert scale), ‘Professional HL regarding ECAP’ (four items, five-point Likert scale), ‘HL-responsive advice’ (seven items, five-point Likert scale), ‘Perception of midwifery work in regard to providing advice on ECAP in an HL-responsive way’ (eight items, five-point Likert scale) were 0.81, 0.78, 0.77, and 0.76, respectively. These values suggest that the internal consistency of the respective scales is acceptable, as they are all above 0.7.

## DISCUSSION

This study describes the process of developing a comprehensive questionnaire to assess the current midwifery practice in relation to providing advice on ECAP in a HL-responsive way and to identify persistent barriers. All 64 items were perceived as relevant and acceptable within the professional context of midwifery. In terms of content, the questionnaire provides a valid representation of current practice and insight into persisting barriers and facilitators despite minor adaptions. Midwives found the questionnaire to be complete and comprehensive for the topic. The piloting indicated acceptability and comprehensibility as well as internal consistency with all relevant items receiving a value >0.7 when calculating Cronbach’s alpha. Additionally, it provided valuable insights into the recruitment of participants.

The questionnaire presented here is based on a large qualitative study. This seems to be quite a strength, as it allows to explore aspects deemed important by the target group. Furthermore, eight cognitive interviews were conducted to ensure that the content of the questionnaire is relevant, comprehensible and provides the response options considered important by the target group. However, the voluntary participation may have introduced a selection bias, as all participants except one had completed higher education. This may be advantageous, as the participants were able to provide critical insight. However, it is possible that midwives with lower levels of education would have found some items less comprehensible.

The questionnaire was only available online and hence, midwives who were reluctant or inexperienced with digital formats may thus have been excluded from the sample. However, we consulted with midwives in practice beforehand to ascertain whether a paper version of the questionnaire would be required. The general response was that this would only have a limited effect on the response rate, as it was considered more onerous to complete a paper questionnaire and return it, than to use an online version.

It is possible that midwives with a special interest in allergy prevention were more inclined to respond to the questionnaire. Midwives with limited or insufficient knowledge of ECAP may have been hesitant to complete the questionnaire due to concerns about being tested and demonstrating a lack of knowledge.

Although previous studies have developed questionnaires on HL of health professionals^24-27^ and HL-responsive advice by health professionals^17^, to our knowledge, our study is the first to focus on practices, barriers and facilitators for midwives in relation to providing advice on allergy prevention in a HL-responsive way. In contrast to Schaeffer et al.^24^, our objective was not to assess the HL of midwives themselves, but rather to examine how they support the HL of parents during consultations. Future research may benefit from combining these two approaches. This could mean, firstly, to measure midwives’ professional HL, for example by using the questionnaire from Schaeffer et al.^24^, and, secondly, continue by collecting data on their HL-responsive strategies when providing advice. Thereby, potential relationships between HL and the ability to provide HL-responsive advice could be assessed. Other studies have placed more emphasis on the identification and communication with patients with limited HL^28,29^, which is a notable difference from our approach. Our interest is to understand the general attitude towards HL-responsive advice on ECAP with all families.

Some of our findings need further discussion. In our previous qualitative interview study, it became clear that midwives had very different perceptions and oftentimes only vague ideas of HL^15^, e.g. being responsible for one’s own health or focusing only on understanding health information. When explicitly asked, others stated that they were not familiar with the term. The German translation of HL (Gesundheitskompetenz) encompasses a very broad concept, which differs from the historically more narrowly defined English term health literacy (with its origin in the functional aspect of being able to read and write)^30^. It was therefore challenging to address midwives’ familiarity with HL appropriately when developing the questionnaire, i.e. we assumed that simply asking whether midwives were familiar with HL could have led to confusion about what midwives actually associate with HL. Therefore, we piloted a version with an open-ended question, asking participants to define HL. The results were very heterogeneous. In a second version, we included a question about familiarity with the definition of HL, and provided the definition of HL^14^ as in the study of Schaeffer et al.^24^. However, our response options differed slightly because we used a different German word for ‘being familiar with something’. This may explain why our pilot study results differ from those of Schaeffer et al.^24^ who concluded that 34.4% of doctors and 38.1% of nurses were at least somewhat familiar with this definition. In contrast, 66% of our sample responded that they were at least somewhat familiar. This discrepancy between the professions is noteworthy and warrants further investigation.

This also relates to the generalizability of the questionnaire. It may be difficult to apply the questionnaire to other populations, as midwives in Germany provide comprehensive in-home care after the birth of a child, which is a rather unique consultation situation that differs significantly from other health professions. Further research is also needed to investigate whether this questionnaire based on a German qualitative study with midwives is applicable in other countries, as there may be differences within the healthcare system regarding midwifery care.

The recruitment of participants for a survey is challenging, especially when it comes to health professionals with a high workload^31^. The questionnaire was distributed via the associations of midwifery in Saarland and Berlin, with 270 members in Saarland and 1020 members in Berlin. The combined response rate of 5.7% was relatively low. However, in Saarland, where the members of the association of midwifery received an individual E-Mail with an invitation and a link to participate, the response rate was 13%. In Berlin, the call for participation was included in a newsletter among other topics and the response rate was only 1.3%. A review by Asch et al.^32^ focusing on problems in recruiting community-based physicians for health service research yielded similar findings. The participation rate was very low when there was no personal contact between recruiter and possible participants (2.7–6%), and considerably higher after personal contact (via telephone 75%, after a personal meeting 91%). In order to facilitate nationwide application, a comprehensive recruitment strategy to increase the return rate and obtain comprehensive understanding of midwifery practice seems warranted^33^. Personalized approaches need to be considered; however, they need to be feasible and not overburdening the research team. Furthermore, the use of social media needs to be reflected upon, especially since the midwifery associations differ significantly in terms of communication with their members (Saarland sends an E-Mail to all members, Berlin uses SharePics on Instagram) and requested for the provision of different materials in order to reach their members efficiently.

## CONCLUSIONS

A 64-item questionnaire was developed by applying the findings of a previously conducted qualitative study to theoretical frameworks. Via a mixed-methods approach, comprising cognitive interviews and pilot testing in two German states, we demonstrated that the questionnaire is content valid, comprehensible and acceptable to the target group, and that the response rates are adequate. Following minor adaptations, the questionnaire can now be employed as a survey instrument to collect data on current practices, barriers, and facilitators of providing advice on ECAP by midwives on a larger scale.

## Supplementary Material



## Data Availability

The datasets generated during and/or analyzed during the current study are available from the corresponding author on reasonable request.
