# Supplementary material for: Development, content validation and piloting of a questionnaire on how midwives in Germany provide advice on early childhood allergy prevention in a health literacy responsive way
Source: Eur J Midwifery. 2026 Feb 18;10:10.18332/ejm/215910. doi: 10.18332/ejm/215910 (PMC12915384; doi:10.18332/ejm/215910)
Supplement: Supplementary file 1 [file EJM-10-04-s1.pdf]

Table 3 Descriptive Analysis of the questionnaire pilot in Saarland and Berlin in March and July 2024.

	Min	Max	Mean	SD[1]	Yes	No	no answer (no)	missing (no)	Scores (%)
**Question 1: I assess the allergy risk of children…**									
…for all families									26 (44,1%)
… for families with pre-existing allergies									19 (32,2%)
…only on request by the parents									8 (13,6%)
…not at all									6 (10,2%)
…no answer									0
**Question 2: I assess the allergy risk of children… (Range 1-5)**									
…verbally	2	5	4,3	0,8					
…in writing, e.g. questionnaire	1	5	1,7	1,2					
…on the basis of existing documents, e.g. maternity record, medical check-up booklet of the child, …	1	5	3,9	1,2					
**Question 3: I find the following topics important when advising parents on allergy prevention (Range 1-5)**									
breastfeeding	3	5	4,9	0,4					
formula, milk-based foods	1	5	4,2	1					
introduction of complementary food	2	5	4,5	0,8					
ingredients and additives in food	2	5	4,1	0,9			1		
personal hygiene, e.g. soap, creams	2	5	4,3	0,9					
environmental hygiene, e.g. cleaning and disinfection of objects,	1	5	3,8	1,1					

[1] Standard Deviation

	Min	Max	Mean	SD[1]	Yes	No	no answer (no)	missing (no)	Scores (%)
detergents, air fresheners									
contact materials, e.g. baby carriages, toys, clothing	1	5	3,3	1,1					
mould and other harmful substances	1	5	4	1,1			1		
contact with nature, playing/crawling outside	1	5	4,2	1					
pets	1	5	3,5	1,2			2		
smoking	2	5	4,7	0,7					
**Question 4: I agree with the following statements on allergy prevention (Range 1-5)**									
Babies should be exclusively breastfed for the first 4-6 months	3	5	4,8	0,6					
Complementary food should be introduced from the beginning of the fifth month and at the latest from the beginning of the seventh month of life with continued breastfeeding	1	5	3,9	1,2					
Boiled chicken eggs and fish should not be introduced in the first year of life	1	5	2,5	1,5					
Pregnant women should follow a special diet to reduce the risk of allergies in the new-born	1	5	1,8	1					
Children at risk of allergies should also be vaccinated in accordance with Standing Committee on Vaccination recommendations	1	5	3,4	1,3			5		
A cat should not be acquired when children	1	5	2,7	1,2			5		

	Min	Max	Mean	SD[1]	Yes	No	no answer (no)	missing (no)	Scores (%)
are at risk of developing allergies									
All children who are not breastfed should receive hypoallergenic formula	1	5	2,1	1,2					
A caesarean section increases a child's risk of allergies	1	5	3,9	1,1			2		
**Question 5a: Are you familiar with the following definition of Health Literacy: Health literacy is the knowledge, motivation and ability to find, understand, evaluate and apply health information.**									
Familiar									19 (32,2%)
More or less familiar									12 (20,3%)
Partly familiar									3 (5,1%)
Somewhat familiar									8 (17%)
Not familiar at all									5 (8,5%)
**Question 5b: I understand Health Literacy as:**									
**Question 6: I find it easy to: (Range 1-5)**									
…inform myself about allergy prevention	1	5	3,5	1				7 (11,9%)	
…recognize whether parents understood what I said	2	5	4,3	0,8				7 (11,9%)	
…assess the Health Literacy of parents	1	5	3,9	0,9				7 (11,9%)	
…pass on information on allergy prevention to parents in an understandable way	1	5	4	0,8				7 (11,9%)	
**Question 7: I know… (Range 1-5)**									
...the national guideline on allergy prevention (%)					33 (55,9%)	17 (28,8%)	2 (3,4%)	7 (11,9%)	
...the exact contents of the national guideline on allergy prevention (%)					17 (28,8 %)	31 (52,5%)	4 (6,8%)	7 (11,9%)	

	Min	Max	Mean	SD[1]	Yes	No	no answer (no)	missing (no)	Scores (%)
...screening tools to assess the health literacy of parents, e.g. questionnaires (%)					12 (20,3%)	37 (67,7%)	3 (5,1%)	7 (11,9%)	
...the teach-back method (%)					30 (50,8%)	20 (33,9%)	2 (3,4%)	7 (11,9%)	
**Question 8: When I advise parents on allergy prevention... (Range 1-5)**									
…I present scientific studies for explanation and discuss these directly with the parents	1	4	2,5	1				7 (11,9%)	
...I talk to parents about the national guideline on allergy prevention	1	5	2,4	1,3			2	7 (11,9%)	
...I explain in my own words what can be done to prevent allergies	1	5	4	0,9				7 (11,9%)	
....I don't talk explicitly about allergy prevention, but about topics such as breastfeeding, nutrition, etc. and tell the parents how best to do this	1	5	3,8	1,1			1	7 (11,9%)	
**Question: 9: When I meet parents for the first time, I assess their health literacy by… (Range 1-5)**									
...my first impression of the family (appearance, parent-child interaction, living environment)	2	5	3,7	0,9			2	7 (11,9%)	
...the conversation with parents (language, questions asked)	2	5	3,9	0,9			1	7 (11,9%)	
...the parents' educational qualifications / occupation	1	5	2,7	1,1				7 (11,9%)	
...using a questionnaire	1	5	1,6	1			2	7 (11,9%)	
**Question 10: When I talk to parents… (Range 1-5)**									

	Min	Max	Mean	SD[1]	Yes	No	no answer (no)	missing (no)	Scores (%)
...I adapt my language to the other person	1	5	4,5	0,8				7 (11,9%)	
...I always explain medical terms	3	5	4,5	0,6				7 (11,9%)	
...I summarize the parents' questions again in my own words to make sure that I have understood them correctly	1	5	3,6	1				7 (11,9%)	
...at the end, I repeat what has been discussed	1	5	4	1				7 (11,9%)	
...I have the parents repeat what I have told them	1	5	2,4	1				7 (11,9%)	
...I use pictures and objects to explain health topics	1	5	2,8	1				7 (11,9%)	
...to conclude, I emphasize the three most important points of the consultation	1	5	3,2	1,1				7 (11,9%)	
**Question 11: I talk to parents about… (Range 1-5)**									
...where and how they can find reliable sources of information on health topics, e.g. on early childhood allergy prevention	1	5	3,2	1,1				7 (11,9%)	
...how they can decide whether a health information is reliable	1	5	2,6	1,1			1	7 (11,9%)	
**Question 12: I agree with the following statements about my work as a midwife: (Range 1-5)**									
As a midwife, I can have significant influence on allergy prevention	3	5	4,3	0,7				7 (11,9%)	
I have enough time during the consultation	1	5	2,7	1,3				7 (11,9%)	

	Min	Max	Mean	SD[1]	Yes	No	no answer (no)	missing (no)	Scores (%)
to talk about allergy prevention									
I come into contact with families early enough to impact on allergy prevention	1	5	3,8	1				7 (11,9%)	
I have materials to give parents good advice on allergy prevention	1	5	2,6	1,1				7 (11,9%)	
I see it as my job as a midwife to advise parents on allergy prevention	1	5	3,9	1,1				7 (11,9%)	
As a midwife, I have the opportunity to improve the health literacy of parents	3	5	4,3	0,7				7 (11,9%)	
I have enough time to adapt to the parents and their information needs	1	5	2,9	1,2				7 (11,9%)	
I think it is important to take parents' health literacy into account when giving advice	2	5	4,4	0,8			1	7 (11,9%)	
**Question 13: To provide parents with information on early childhood allergy prevention, I would like to have:**									
**Question 14: To better assess and consider parental HL, I would like to have:**									
**Question 15: I would like to receive further training on the topic of health literacy in the following format (multiple choice possible)**									
in person training									19 (32,2%)
online training courses									38 (64,4%)
books									8 (13,6%)
scientific publications									13 (22%)
journals									12 (20,3%)
brochures									21 (35,6%)

	Min	Max	Mean	SD[1]	Yes	No	no answer (no)	missing (no)	Scores (%)
newsletters (for example from the associations of midwifery)									21 (35,6%)
information from scientific services of the industry (e.g. food manufacturers)									5 (8,5%)
websites									13 (22%)
apps									8 (13,6%)
podcasts									14 (23,7%)
none of the above									3 (5,1%)
no answer									7 (11,9%)
other: ________________									0
**Question 16: I would like to receive further training on the topic of allergy prevention in the following format (multiple choice possible)**									
in person training									25 (42,4%9
online training courses									41 (69,5%)
books									10 (16,9%)
scientific publications									16 (27,1%)
journals									19 (32,2%)
brochures									27 (45,8%)
newsletters (for example from the associations of midwifery)									21 (35,6%)
information from scientific services of the industry (e.g. food manufacturers)									6 (10,2%)
websites									16 (27,1%)
apps									11 (18,6%)
podcasts									13 (22%)
none of the above									2 (3,4%)

	Min	Max	Mean	SD[1]	Yes	No	no answer (no)	missing (no)	Scores (%)
no answer									7 (11,9%)
other: ________________									0
**Question 17: I would like information on allergy prevention to be passed on to parents in the following form (multiple choice possible)**									
Scientific publications prepared for parents									26 (44,1%)
brochures									43 (72,9%)
videos (e.g. YouTube)									28 (47,5%)
websites									19 (32,3%)
podcasts									18 (30,5%)
apps									16 (27,1%)
journals/magazines									7 (11,9%)
posters									10 (16,9%)
none of the above									1 (1,7%)
no answer									7 (11,9%)
other:________________									1 (1,7%)

© 2026 von Sommoggy J. et al.
